# Anesthesia and cancer

**DOI:** 10.1590/1806-9282.2024S102

**Published:** 2024-06-07

**Authors:** Plínio da Cunha Leal, Marcos Antônio Costa de Albuquerque, Luis Antonio dos Santos Diego, Maria Ângela Tardelli

**Affiliations:** 1 Universidade Federal do Maranhão, Santo Domingo Hospital, Teaching and Training Center, Scientific Department of the Brazilian Society of Anesthesiology – São Luís (MA), Brazil.; 2 Scientific Department of the Brazilian Society of Anesthesiology, Scientific Department of the Latin American Confederation of Anesthesiology – Aracaju (SE), Brazil.; 3 Universidade Federal Fluminense, Brazilian Society of Anesthesiology – Rio de Janeiro (RJ), Brazil.; 4 Universidade Federal de São Paulo, Brazilian Society of Anesthesiology – São Paulo (SP), Brazil.

## INTRODUCTION

**Figure 1:**
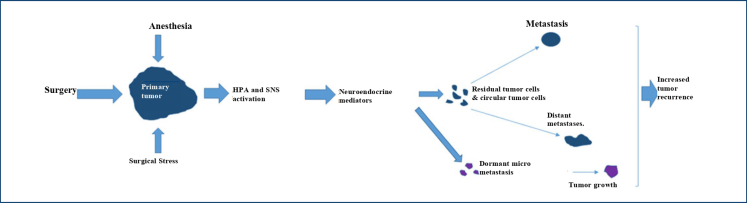
Hypothesis to explain cancer metastasis and recurrence caused by surgery and perioperative anesthetic-induced immunosuppression. Surgery, anesthesia, and analgesia stimulate the hypothalamic-pituitary-adrenal axis and the sympathetic nervous system during the perioperative period. Activated neuroendocrine mediators lead to increases in several soluble immunosuppressive factors that promote tumor progression and metastasis, resulting in increased cancer recurrence. Adapted from Kim^1^.

Major surgeries have an influence on the neuroendocrine system [hypothalamic-pituitary-adrenal (HPA) axis and sympathetic nervous system (SNS)], leading to cytokine-mediated stress responses that cause immunosuppression. During surgical procedures, tumor cells are released, and tumor emboli are disseminated. Hence, the surgery itself appears to be associated with an increased risk of cancer metastasis and recurrence^1^ (Figure 1).

## PERIOPERATIVE IMMUNOSUPPRESSION

Th1-type responses, including CD8^+^ T lymphocytes and natural killer (NK) cells, are required for immunity against tumor growth. However, the dominant Th2 status, yet not Th1, develops in cancer patients. In this context, surgical stress further induces Th1/Th2 balance toward Th2^2^-type immune responses.

The main causes of responses toward immunosuppression in surgical patients relate to the neuroendocrine stress exerted by SNS and HPA axis activation^1^.

The immune system is innervated with sympathetic nerve fibers and catecholamines (adrenaline and noradrenaline) released from the nerve terminal, binding to b2-adrenergic receptors expressed in T cells, NK cells, and macrophages^1^.

These interactions between catecholamines and b2-adrenoreceptors increase intracellular cyclic adenosine monophosphate (cAMP), inhibit NK cell activity, and polarize T cells and macrophages in Th2 cytokine production, leading to a shift toward the Th2 response, although catecholamines are known to mobilize b2-adrenoreceptors. Therefore, sympathetic activation during surgery suppresses antitumor immunity^1^.

HPA axis activation leads to the increased production of adrenocorticotropic hormone (ACTH) in the pituitary gland, releasing glucocorticoids from the adrenal glands. The interaction between glucocorticoids and receptors expressed on immune cells prevents the production of Th1 cytokines on macrophages and T cells, promoting Th2^1^ polarization.

## HYPOTENSION, HYPOVOLEMIA, AND HYPOXIA

Hypotension and hypovolemia activate the SNS and HPA axes; they also cause decreased tissue perfusion and cellular hypoxia, which induce increased adhesion molecule expression in the vascular endothelium, initiating a systemic inflammatory response that results in reduced Th1 activity. Hypoxia impulses generate hypoxia-induced factor (HIF) activation in the immune cells and tumor cells^3^.

Increasing HIF production in T cells induces a change from a Th1 to a Th2 phenotype by increasing interleukin-10 (IL-10) production and decreasing IFN-γ, as well as stimulating Treg cell differentiation and proliferation. Expression of HIF on tumor cells promotes tumor cell proliferation and induces angiogenic factor^3^ secretion.

## HYPOTHERMIA

*In vitro* experimental studies demonstrate that monocytes incubated at low temperatures reduce human leukocyte antigen-DR isotype (HLA-DR) antigen expression and increase tumor necrosis factor-α (TNF-α) and IL-10 release, generating chronic inflammation. *In vivo* animal studies have revealed that hypothermia suppresses NK cell activity and increases tumor metastasis risk^4^.

## HYPERGLYCEMIA

Acute perioperative hyperglycemia inhibits glucose-6-phosphate dehydrogenase, the enzyme responsible for forming nicotinamide adenine dinucleotide phosphate, suppressing monocyte and neutrophil functions. The degree of hyperglycemia required to impair phagocytosis is about 200 mg dL^-1^. However, hyperglycemia can evoke leukocyte adhesion-triggered microvascular inflammation in the endothelium through the generation of adhesion molecules. Microvascular inflammation, depending on high glucose- and NF-κB activation-associated increased osmolarity, leads to increased inflammatory cytokine production in addition to HPA axis activation. Thus, insulin can reduce the levels of these cytokines and inhibit the NF-κB pathway in monocytes, playing an anti-inflammatory role^5^.

## BLOOD TRANSFUSION

Allogeneic blood transfusion is known to cause transfusion-related immunomodulation (TRIM). TRIM can be mediated by allogeneic mononuclear cells, soluble mediators derived from white blood cells, and allogeneic plasma-soluble HLA peptides. However, removal of leukocytes from allogeneic blood failed to reduce TRIM because transfusion of packed red blood cells also suppresses immunity in patients receiving allogeneic blood transfusions^6^.

While the mechanisms by which allogeneic blood transfusion suppresses recipients’ immunity remain unclear, deleucotized donor red blood cells may have direct suppressive effects. Red blood cells contain constituent substances such as metabolically active arginase, an enzyme of the urea cycle that is expressed in cells throughout the body and limits the availability of arginine, which is an amino acid needed for T cell proliferation and expression of the functional ς chain of cytotoxic lymphocytes. Therefore, arginase-mediated arginine depletion can strongly suppress the function of T-cell receptors^7^.

In addition, allogeneic blood transfusion-related immunosuppression is mediated by the induction of Treg, which can suppress CD4^+^ and CD8^+^ T cells and inhibit dendritic cell function. Therefore, for reducing both transfusion and blood loss in surgeries, the use of cell saver and erythropoietin is worthy of consideration^7^.

## NUTRITIONAL STATUS

Preoperative malnutrition, present with some frequency in cancer patients, leads to immunosuppression. Therefore, some authors advocate the practice of the so-called "immunonutrition", in which the administration of arginine and omega-3 fatty acids may favor Th1 polarization and thus have a beneficial effect on these patients^8^.

## DRUGS

### Inhaled anesthetics

A study investigated the effects of isoflurane on the expression of tumor markers, including insulin-like growth factor (IGF-1) and proliferative capacity in ovarian cancer cells, and demonstrated that isoflurane significantly increased IGF-1 receptor expression, cell cycle progression, and cell proliferation in ovarian cancer cells. It also showed increased expression of the angiogenic markers, namely, vascular endothelial growth factor (VEGF) and angiopoietin-1. Cancer cell migration after exposure to isoflurane has been associated with an increased production of metalloproteinases 2 and 9, enzymes that ease local dissemination of tumor cells^9^. A small study with 40 patients presenting for colon cancer surgery showed that serum levels of pro-angiogenic VEGF-C factors and transforming growth factor beta-1 increased significantly in patients receiving inhalational anesthesia versus propofol-epidural anesthesia^10^.

Another study evaluating the response of glioma stem cell exposure to varying durations and concentrations of sevoflurane compared to controls showed increased cancer cell proliferation and a capacity for self-renewal following sevoflurane use^11^.

Kvolik et al., investigated the cytotoxic and antiproliferative effects of sevoflurane on different *in vitro* human cancer cell lines and found that the apoptotic rate significantly increased 24 h after anesthesia and was associated with the increased expression of the p53 and caspase-3 genes in colon cancer cells. They also noted a decrease in laryngeal cancer cell expression, suggesting any potential beneficial effect of this volatile agent on increasing cell apoptosis in this cancer and that it may be tumor cell line-dependent^12^.

Although conflicting evidence remains on the potential deleterious effects of volatile agents based on the in vitro study evidence to date, there is insufficient evidence to justify avoiding these agents in cancer patients^13^.

### Nitrous oxide

The immunosuppressive effect of nitrous oxide, mediated through selective inhibition of methionine synthase and, therefore, purine and thymidylate synthesis, causes macrophage and NK cell function depression^14^. The ENIGMA-II trial found that the use of nitrous oxide did not interfere with cancer recurrence or mortality^15^.

Another study with a specific focus on nitrous oxide and cancer evaluated the recurrence rate of colon cancer in a randomized trial with 204 patients undergoing 65% nitrous oxide or oxygen concentration during surgery and found a similar recurrence rate in both groups^16^.

### Propofol

Some of the direct effects of propofol include inhibition of proliferation, migration, invasion, and induction of apoptosis based on micro-RNA changes and influence on signaling pathways such as inhibition of mitogen-activated protein kinase (MAPK), nuclear factor-κB (NF-κB), and HIF-1α^17^. On the contrary, propofol has been described to activate erythroid nuclear factor-related factor 2 (Nrf2) in bladder cancer, which leads to apoptosis inhibition. Propofol indirectly interferes with tumor progression by increasing chemosensitivity and maintaining immune function. Increased chemosensitivity was found for trastuzumab in breast cancer, paclitaxel and cisplatin in ovarian cancer, and gemcitabine in pancreatic cancer. Propofol conserves immune function compared to sevoflurane, which suppresses T1-lymphocytes in cervical and colorectal cancer^18^.

However, another *in vivo* study showed a depletion of tumor-associated macrophages from the tumor microenvironment and an upregulation of immune checkpoint-programmed death ligand-1 (PD-L1) by sevoflurane in melanomas, indicating a possible positive effect of sevoflurane in combination with the checkpoint-programmed death-1 (PD-1)^19^ inhibitor.

Several retrospective analyses indicate a beneficial effect of propofol compared to inhaled agents. A meta-analysis carried out by Jin et al.^20^ summarized 12 studies with an overall mortality hazard ratio of 0.73% [95% confidence interval (CI) 0.60–0.89] for total intravenous anesthesia (TIVA). However, when divided into subgroups of different types of cancer, only a statistical analysis of breast and colorectal cancer could be run, showing a positive trend for TIVA in colorectal cancer but not in breast cancer. The limitations of this study are numerous: retrospective design, lack of statistical strength, and uncertain control of confounding factors. Still, a large cohort study in Japan (166,966 inhalational anesthesia, 29,337 TIVA) showed no difference in survival compared to any digestive tract cancer surgery. There was, however, a slight advantage in recurrence-free survival for TIVA upon analyzing instrumental variables (95%CI 0.87–0.98; p=0.01)^21^.

Few well-designed clinical trials have prospectively investigated the use of propofol and tumor recurrence. A large multicenter randomized controlled trial (n=2108) compared recurrence rates (7-year follow-up) after curative breast cancer resection and found no difference between a paravertebral block combined with propofol and general anesthesia with sevoflurane and opioids^22^.

### Ketamine

Ketamine as an anesthetic or at higher doses (up to 80 mg kg^-1^) has been shown to suppress NK cell activity, possibly via sympathetic activation. Additionally, low-dose ketamine as an adjuvant to general anesthesia reduced inflammatory responses and pain after cancer surgery, which could be advantageous for mitigating NK cell activity suppression. Ketamine can exert a direct influence on NK cell activity as its use leads to N-methyl-D-aspartate (NMDA) receptor activation suppression and subsequent changes in intracellular calcium and reactive oxygen species^23^.

A study by Duan et al.^24^ demonstrated that ketamine decreased intracellular calcium levels, leading to a reduction of VEGF1 expression and cell migration. It concluded that the antitumor effect of ketamine can be achieved by blocking the NMDA receptor. A meta-analysis also showed the anti-inflammatory property of ketamine on cytokines, especially IL-6^25^.

In another study carried out by Forget et al.^26^ which evaluated the use of analgesics on tumor recurrence after mastectomy, the use of ketamine was not associated with an improvement in cancer patient outcome.

### Alpha agonists

Despite their frequent use as sedatives and analgesic agents, very few studies focus on the effects of α-2 adrenoceptor agonists on cancer. Given the overall pro-tumor effects of catecholamines, it can be postulated that agents that similarly activate adrenoceptors should also promote carcinogenic effects. On the other hand, a small, randomized trial with patients undergoing radical gastrectomy for dexmedetomidine or saline infusion demonstrated that dexmedetomidine resulted in reduced levels of catecholamines and pro-inflammatory cytokines, suggesting a potentially beneficial antitumor effect^27^.

While animal studies have shown potential for promoting cancer recurrence and metastasis due to their role in facilitating angiogenesis, thus leading to metastasis, randomized human studies have not shown conclusive results^28^.

Evidence suggests that dexmedetomidine may reduce the degree of immune function suppression and keep the number of CD3+ cells, NK cells, the CD4+/CD8+ ratio, and the Th1/Th2 ratio stable by decreasing the level of pro-inflammatory cytokines (IL-6 and TNF-α) during cancer operations. However, dexmedetomidine exhibits different roles in cell biology behavior depending on the types of cancer cells. Therefore, this is still a new area that needs further exploration.

### Opioids

There is conflicting evidence from experimental studies investigating the role of opioids in tumor growth and metastases. Several animal studies have found that some opioids promote immunosuppression and, in turn, postoperative tumor recurrence, with effects on immune function varying between different types of opioids. Namely, morphine has been shown to suppress NK cell cytotoxicity and T cell proliferation. However, a few studies contradict these findings by proposing that morphine has antitumor effects. Similarly, fentanyl has shown the inhibition of NK cells and the promotion of lymphocyte and macrophage apoptosis in several laboratory studies. Still, a recent retrospective cohort study with 1,679 patients with stage I-III colorectal cancer showed no association between fentanyl and oncological or prognostic outcomes. Alternatively, tramadol has been shown to have immunostimulatory properties by increasing the cytotoxicity of NK^29^ cells.

A special interest emerged in methadone, which has been shown to increase the apoptosis and chemosensitivity of *in vitro* and *in vivo* leukemic and glioblastoma cells through a reduction of cAMP, which leads to caspase activation. However, these preclinical findings have not yet been found in well-designed clinical studies, and the adverse effects of methadone, especially in pain-free patients, should be considered with caution^30^.

There is also evidence that mu opioid receptors (MOR) are overexpressed in certain cancers. As a consequence, opioid binding in MOR directly promotes cancer cell growth via growth factor-induced receptor signaling and angiogenesis potentiation. A lung sample study with 34 lung cancer patients demonstrated a twofold increase in MOR expression in patients with metastatic lung disease. Clinical studies further support the role of MOR in cancer progression. In a retrospective study with 113 patients with prostate cancer, MOR overexpression was associated with reduced overall survival and progression-free survival, especially in those with metastatic disease. In line with these results, two randomized clinical trials showed that treatment with methylnaltrexone (a MOR antagonist) is associated with increased overall survival in terminal cancer patients^31,32^.

Overall, the role of opioids in facilitating tumor recurrence and metastasis is variable and influenced by opioid type, dosage, and form of administration. More controlled, randomized, and prospective studies are still needed for higher-quality clinical evidence.

## LOCAL ANESTHETICS

If administered epidurally, local anesthetics are partially absorbed into the bloodstream, reaching concentrations of 1–10 μM. This concentration of local anesthetics also reaches tumor cells. *In vitro* data showed a dose-dependent antiproliferative effect of local anesthetics in various cancer types. For example, inhibition of migration, invasion, and progression of colorectal cancer cells in response to lidocaine (10 μM), ropivacaine (10 μM), and bupivacaine (1 mM). Similar results were found in gastric cancer. Low bupivacaine concentrations (10 μM) reduced the migration of gastric cancer cells, while high concentrations (1 mM) also increased apoptosis^33^.

The most recent evidence points to a possible synergistic effect of LA along with chemotherapy. *In vitro*, lidocaine appears to have a potentiating effect on cisplatin chemotoxicity through demethylation of retinoic acid receptor beta 2 (RAR beta 2), located in the cell nucleus, and the Ras association domain-containing tumor suppressor 1 (RASSF1) protein in breast cancer cells. In another recent study by Chamaraux-Tran et al.^34^ lidocaine demonstrated a direct cytotoxic effect on *in vitro* breast cancer cells and in an *in vivo* mouse model.

LA can also induce apoptosis in cancer cells by activating caspases and regulating the MAPK signaling pathway. The inhibitory effect of lidocaine on Src tyrosine-protein kinase indicates that systemically administered local anesthetics can potentially prevent tumor cell metastasis^35^.

A retrospective analysis of intraoperative IV lidocaine use in pancreatic surgery (n=915 in each group) revealed an improvement in overall survival after 1 (68% vs. 62.6%, p<0.001) and 3 years (34.1% vs. 27.2%, p=0.011)^36^. A controlled, randomized, prospective study identified a reduction of myeloperoxidase, histone H3, and matrix metalloproteinase MMP3 via intraoperative infusion of IV lidocaine during breast cancer surgery. These findings support the hypothesis of an antimetastatic effect of lidocaine^37^.

Another study compared the rate of breast cancer recurrence after curative surgery in more than 2,000 patients who received propofol-based anesthesia in combination with a paravertebral nerve block or general anesthesia with sevoflurane and an opioid-based analgesic regimen. There was no difference in relation to the primary outcome between these two groups^38^.

Current evidence supports the use of intraoperative lidocaine IV infusion as a supplement in pain therapy when epidural anesthesia is not possible or desired. In addition, the hypothesis of lidocaine having an anticancer effect has been formulated, but benefits in terms of survival and recurrence rates have not yet been demonstrated in prospective randomized clinical trials.

## CONCLUSION

With the increasing number of patients undergoing oncological surgeries and the number of studies suggesting possible long-term effects of the anesthetic technique on tumor growth, there is an increased need for more multicenter studies that can address these issues more clearly.

**Figure 2:**
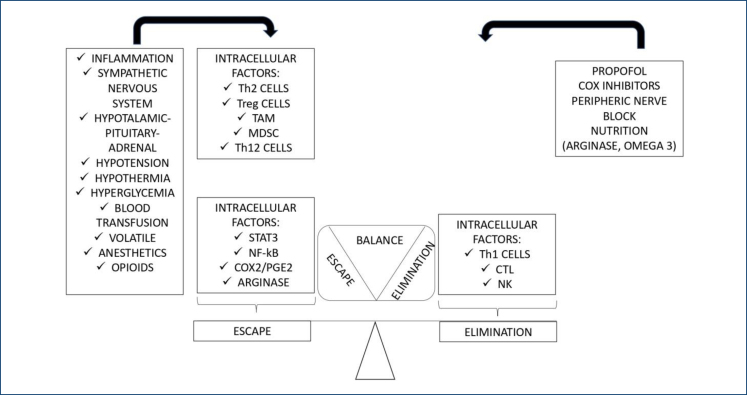
Mechanisms related to TH1 or TH2 polarization and their relationship with factors present in the perioperative period. Adapted from Junqueira et al.^8^. Th2: helper 2 type cells; Treg cells: regulatory cells; TAM cells: macrophage-associated tumor cells; MDSC cells: myeloid suppressor cells; NK cells: natural killer cells; STAT3: signal transducer and activator of transcription 3; NF-κB: nuclear factor kappa light-chain enhancer of activated B cells; COX-2: cyclooxygenase 2; PGE2: prostaglandin 2.

A summary of what was exposed in this article can be seen in Figure 2.
